# *FBXW4* Is Highly Expressed and Associated With Poor Survival in Acute Myeloid Leukemia

**DOI:** 10.3389/fonc.2020.00149

**Published:** 2020-02-27

**Authors:** Qi Han, Qi Zhang, Huihui Song, Yevgeniya Bamme, Chunhua Song, Zheng Ge

**Affiliations:** ^1^Department of Hematology, Zhongda Hospital, School of Medicine, Southeast University, Institute of Hematology Southeast University, Nanjing, China; ^2^International Cooperative Leukemia Group and International Cooperative Laboratory of Hematology, Zhongda Hospital, School of Medicine, Southeast University, Nanjing, China; ^3^Department of Pediatrics, Pennsylvania State University Medical College, Hershey, PA, United States

**Keywords:** *FBXW4*, expression, clinical feature, survival, acute myeloid leukemia

## Abstract

The F-box and WD repeat domain-containing (FBXW) proteins play an important role in ubiquitin proteasome by inducing protein degradation. Ten FBXW proteins have been identified in humans. The functions of FBXW proteins, like FBXW7, have been well-established in many human cancers. However, little is known about their transcriptional expression profiles and relationship with prognosis in acute myeloid leukemia (AML). Here we investigated the roles of FBXW proteins in AML by analyzing their mRNA expression profiles and association with clinical features using data from EMBL-EBI, the Cancer Cell Line Encyclopedia, Gene Expression Profiling Interactive Analysis, and cBioPortal databases. Our results showed that the mRNA level of FBXW proteins were highly detected by microarray in 14 AML cell lines, although there were no obvious differences. The expression of *FBXW4* was significantly higher in AML patients compared with that in normal controls (*P* < 0.01). Patients whose age was ≥60 years old had a higher *FBXW4* expression when compared with those who were <60 years old (*P* < 0.05). Cytogenetic favorable-risk group patients had a much lower *FBXW4* expression than the intermediate- and poor-risk group patients (*P* < 0.0001). Moreover, patients with high *FBXW4* expression exhibited significantly shorter event-free survival (EFS) and overall survival (OS) than those with low *FBXW4* expression (median EFS: 5.3 vs. 10.0 months, *P* = 0.025; median OS: 8.1 vs. 19.0 months, *P*= 0.015). A multivariate analysis indicated that high *FBXW4* expression was an independent risk factor for poor EFS in AML patients who received intensive chemotherapy followed by allo-SCT. In summary, our data suggested that *FBXW4* is aberrantly expressed in AML and high *FBXW4* expression might be a poor prognostic biomarker; future functional and mechanistic studies will further illuminate the roles of *FBXW4* in AML.

## Introduction

Acute myeloid leukemia (AML) is a hematopoietic malignancy originating from myeloid precursors and is the most common acute leukemia in adults (1). AML is still incurable in 60–65% of patients who are 60 or younger due to refractory or relapsed disease. For patients who are older than 60, the prognosis is even worse (2, 3). The rapid development of high-throughput sequencing technology has uncovered a series of genetic alterations which can predict clinical outcomes and lead to potential new therapeutic targets in AML (4–7).

F-box and WD repeat domain-containing (FBXW) proteins have been shown to play a role in ubiquitin proteasome-induced protein degradation. Until now, 10 FBXW proteins have been identified: FBXW1 (BTRC), FBXW2, FBXW4, FBXW5, FBXW7, FBXW8, FBXW9, FBXW10, FBXW11, and FBXW12. All of the FBXW proteins have one F-box domain and unequal amounts of WD repeat domain. The F-box domain is responsible for the assembly of ubiquitin ligase and the WD repeat domain binds to substrate proteins which will be degraded. Of all 10 FBXW proteins, FBXW7 is the one whose function has been studied most thoroughly (8). Under normal physiological conditions, the substrates can bind to FBXW7 after they have been phosphorylated at specific sites (9). Moreover, most of these substrates have been identified as key molecules or transcription factors that play important roles in the regulation of cell growth and cell proliferation, such as cyclin E, MYC, and NOTCH (9–14). Because these substrates are generally considered as proto-oncogene proteins that are widely involved in the tumorigenesis of human cancers, FBXW7 has been recognized as a tumor suppressor for its ability to mediate the degradation of these proteins (9). Importantly, in T-cell acute lymphoblastic leukemia and some solid tumors, many loss-of-function mutations have been identified in *FBXW7*. These mutations influence the interaction of FBXW7 with its substrates and then impair their degradation (15, 16). FBXW2 has also been shown to be a tumor suppressor in lung cancer cells by promoting SKP2 degradation (17).

On the other hand, other F-box and WD repeat domain-containing proteins have been reported to participate in or promote cancer cell growth and proliferation via interaction with different substrates. For example, CUL4A–DDB1–FBXW5 E3 ubiquitin ligase complex can promote non-small cell lung cancer cell growth by mediating DLC1 degradation (18). The proliferation of choriocarcinoma cells can be inhibited by siRNA-induced silencing of *FBXW8* expression. In human colon cancer cells, FBXW8-mediated degradation of cyclin D1 is critical for survival and proliferation (19, 20). These results indicate that F-box and WD repeat domain-containing proteins may have different and complex functions in tumor suppression or tumorigenesis. There have only been a few reports about mRNA expression of FBXW proteins in human cancers and their association with clinical prognosis. *FBXW11* expression is reported to be upregulated in lymphocytic leukemia patients and dramatically decreased in patients after they achieved complete remission (CR) (21). Recent work has shown that *FBXW7* expression was downregulated in T-cell lymphoblastic lymphoma (22), which is in accordance with its tumor suppressor function. Similar expression characteristics of *FBXW7* in human osteosarcoma have also been observed (23). However, the transcriptional expression features and clinical significance of FBXW proteins in acute myeloid leukemia (AML) have not been established. Here we analyzed the mRNA expression characteristics of F-box and WD repeat domain-containing proteins in AML cell lines and AML patients using Cancer Cell Line Encyclopedia (CCLE) and Gene Expression Profiling Interactive Analysis (GEPIA) online databases. Clinical prognostic significances were further examined in members with differential expression between AML patients and normal controls using cBioPortal TCGA database. Lastly, protein–protein interaction network and the potential biological function of FBXW4 in AML were explored by STRING and GeneMANIA databases and Gene Set Enrichment Analysis (GSEA).

## Methods

### EMBL-EBI Dataset

EMBL-EBI (https://www.ebi.ac.uk) is an open-access dataset which provides numerous bioinformatics applications, including gene expression characteristics in human cancer cell lines (23). The F-box and WD repeat domain-containing family members' expression in AML cell lines is analyzed by the EMBL-EBI dataset.

### CCLE Dataset

We explored *FBXW10* and *FBXW12* expression characteristics in AML cell lines using the CCLE dataset. The CCLE (https://www.broadinstitute.org/ccle) dataset is an online tool which provides gene expression data, mutation data, fusion/translocation data, and CpG methylation data for 84,434 genes and 1,457 cell lines freely (24).

### GEPIA Dataset

The expression differences of F-box and WD repeat domain-containing family members between AML patients and normal patients were conducted by GEPIA dataset. GEPIA is a newly developed dataset which provides RNA sequencing information of 9,736 tumors and 8,587 normal samples from the TCGA and the GTEx projects in 33 different types of cancers. The RNA-Seq datasets that GEPIA used are based on the UCSC Xena project (http://xena.ucsc.edu) and are computed by a standard pipeline. Besides gene expression data, GEPIA also provides survival analysis, correlation analysis, and some other advanced bioinformatic analyses (25).

### Patient Data

The RNA expression data (transcripts per million and Z-score), clinical and laboratorial parameters data, *FLT3, TP53, ASXL1*, and *RUNX1* mutation conditions, and survival data of 173 out of 200 newly diagnosed AML patients from the TCGA dataset were downloaded from the cBioPortal dataset (26). Sixteen acute promyelocytic leukemia (APL, M3) patients were excluded from analysis for its' unique molecular signature and much better prognosis than those of other AML patients. Moreover, two patients without specific FAB subtype classification were also excluded from the analysis. Lastly, 155 *de novo* non-M3 AML patients were enrolled to go with further analysis. As a powerful tool, cBioPortal dataset provides a simple and convenient approach to access patient data from TCGA and other datasets (27, 28).

### Bioinformatics Analysis

FBXW4 protein–protein interaction network was predicted by the STRING and GeneMANIA datasets. STRING and GeneMANIA are both frequently used datasets which can provide protein–protein interaction information (29, 30). *FBXW4* co-expression network was analyzed by cBioPortal dataset. Gene Set Enrichment Analysis (gene sets: c2.all.v7.0.symbols.gmt) was performed to explore the potential biological pathways of FBXW4 involved in AML.

### Statistical Analysis

The patients were divided into a high *FBXW4* expression group and a low *FBXW4* expression group [transcripts per million (TPM): 1st quartile vs. TPM: 2nd to 4th quartile; Z-scores ≥1 vs. <1]. All of the statistical analyses were carried out using SPSS 24.0 software (SPSS Inc., Chicago, IL, USA). The relationship between *FBXW4* expression and the clinical and laboratorial parameters was analyzed by chi-square test. Kaplan–Meier method and log-rank test were used to generate the survival curves and analyze the survival difference between the high and the low *FBXW4* expression group patients. Multivariate analyses were performed using Cox proportional hazards model. *P* < 0.05 were considered as statistically significant.

## Results

### Transcriptional Expression Features of FBXW Proteins in 14 AML Cell Lines

**Figure 1:**
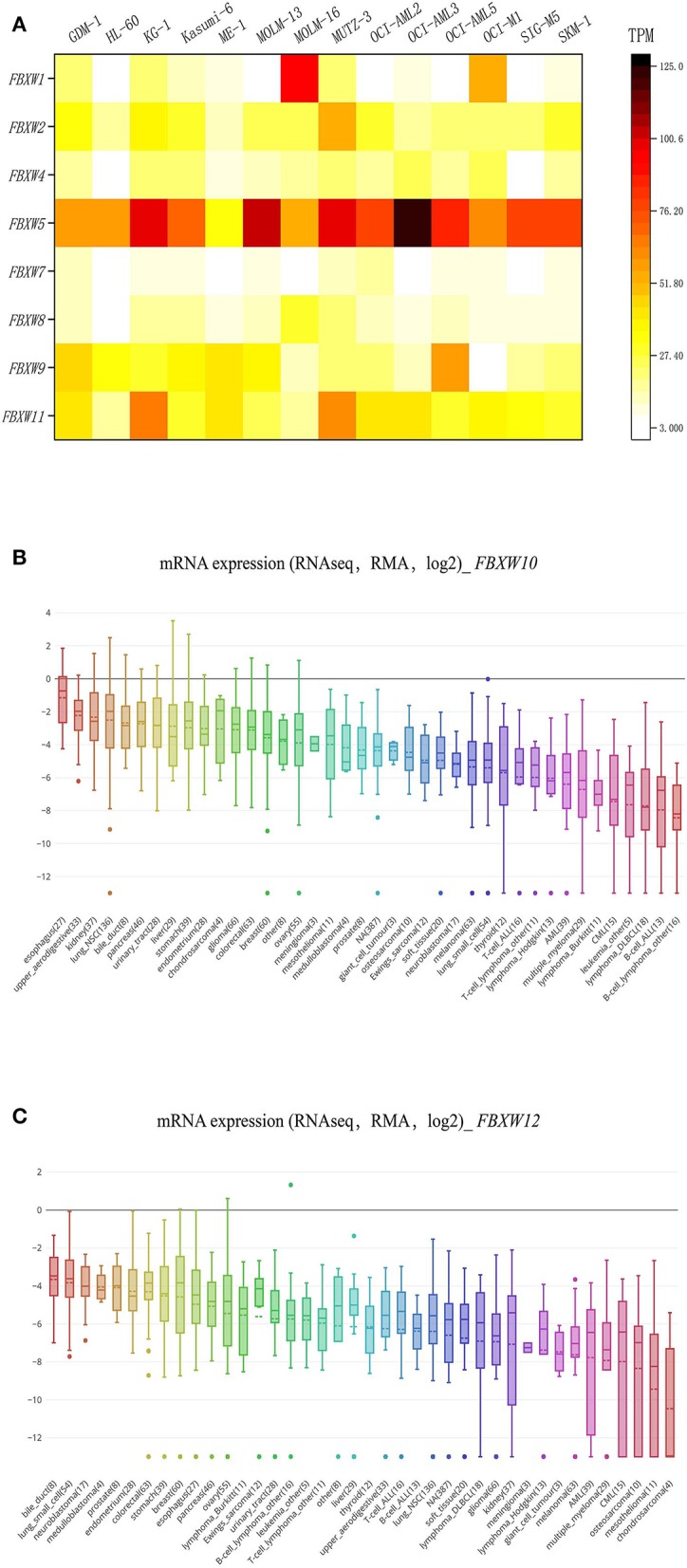
F-box and WD repeat domain-containing family member expression features in AML cell lines. **(A)** Heatmap of *FBXW1, FBXW2, FBXW4, FBXW5, FBXW7, FBXW8, FBXW9*, and *FBXW11* expression in 14 AML cell lines; **(B,C)** expression features of *FBXW10* and *FBXW12* in 30 human cancer cell lines.

We explored the transcriptional expression profiles of FBXW proteins in 14 AML cell lines using the EMBL-EBI database. Results indicated that *FBXW5, FBXW11, FBXW2, FBXW9, FBXW4, FBXW8*, and *FBXW7* are well expressed in the cell lines, although *FBXW5* had the highest expression in the cells. *FBXW1* was specifically highly expressed in the MOLM-16 AML cell line (Figure 1A). EMBL-EBI database has no *FBXW10* and *FBXW12* expression data, but from the CCLE database, we found that they are expressed at a low level in all human cancer cell lines, including AML (Figures 1B,C).

### Transcriptional Levels of FBXW Proteins in AML Patients

**Figure 2:**
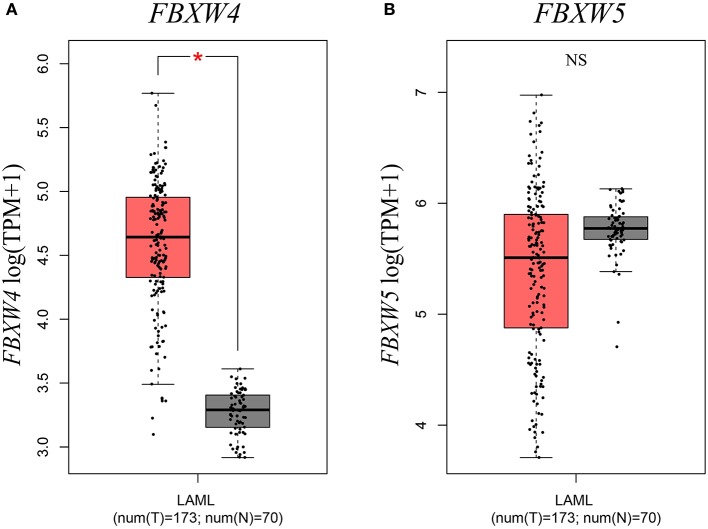
Expression differences of *FBXW4* and *FBXW5* between 173 *de novo* AML patients and 70 normal controls. **(A)** *FBXW4*. **(B)** *FBXW5* (*P < 0.01).

Next, to further understand the transcriptional expression features of FBXW proteins in AML, we investigated their mRNA levels in AML patients with the GEPIA computer tool and the data from the TCGA database. Results showed that the expression of *FBXW4* was significantly upregulated in AML patients compared with that in normal controls (*P* < 0.01) (Figure 2A). Other family members did not exhibit a significant difference in their mRNA levels between AML patients and normal controls (*P* > 0.05) (Figure 2B, Figure S1). Consistent with its expression characteristics in AML cell lines, *FBXW5* mRNA level was higher in AML patients compared with other FBXW proteins (Figure 2B); however, it is also higher in normal controls. Therefore, no significant difference was observed between the AML patients and the normal controls. *FBXW10* and *FBXW12* showed low mRNA levels in both AML patients and normal controls (Figure S1).

### High *FBXW4* Expression Is Associated With Older Age and Poorer Cytogenetic Risk Classification in AML Patients

**Figure 3:**
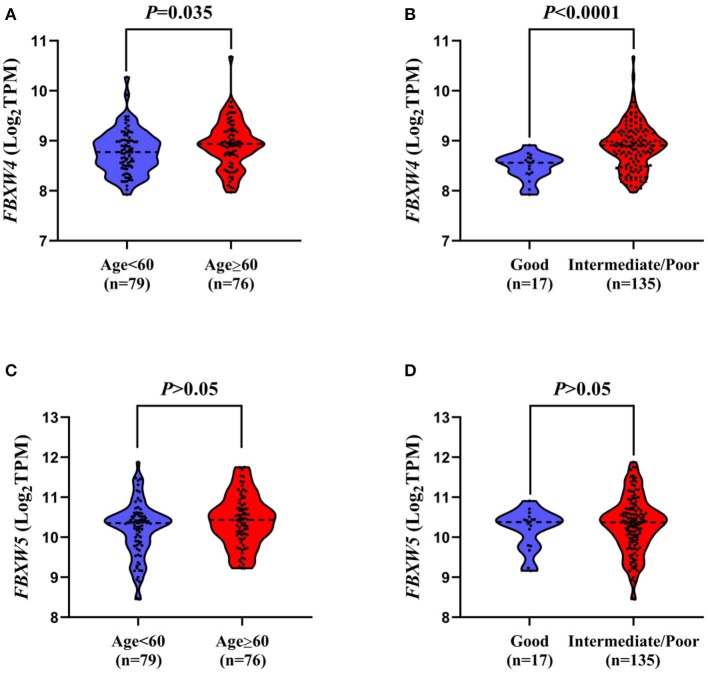
Association of *FBXW4* and *FBXW5* expression with age and cytogenetic risk classification in AML patients. *FBXW4* expression difference between patients ≥60 and <60 **(A)** and patients with good-risk cytogenetic classification and intermediate/poor-risk cytogenetic classification **(B)**. *FBXW5* expression difference between patients ≥60 and <60 **(C)** and patients with good-risk cytogenetic classification and intermediate/poor-risk cytogenetic classification **(D)**.

In order to further explore the association of *FBXW4* expression with clinical features in AML patients, *FBXW4* mRNA (RNA Seq V2 RSEM) expression data and clinical parameters were analyzed with RNA-seq datasets downloaded from cBioPortal TCGA database. We dichotomized the patients into a high *FBXW4* expression group (1^st^ quartile) and a low *FBXW4* expression group (2nd−4th quartile) based on *FBXW4* TPM or a high and low *FBXW4* expression with gene expression *Z*-score ≥1 and Z-scores <1, respectively (Table 1). We found that patients with a high expression of *FBXW4* (Z-scores ≥1) were relatively older than those patients with a low *FBXW4* expression (Z-scores <1) (median: 63.5 vs. 58, *P* = 0.046). Also, patients with age ≥60 years old at diagnosis had higher *FBXW4* expression when compared with patients whose age was <60 years old (*P* = 0.035) (Figure 3A). The relationship between *FBXW4* expression and cytogenetic risk classification was further analyzed. It is shown that patients with favorable risk had much lower *FBXW4* expression compared with patients with intermediate risk and poor risk (*P* < 0.0001) (Figure 3B). No significant differences in *FBXW4* expression were observed with sex, bone morrow blasts, peripheral blood blasts, and *FLT3-ITD, FLT3-TKD*, and *TP53* mutation, ELN risk classification, treatment options (intensive or non-intensive), and allo-SCT (*P* > 0.05) (Table 1). Taken together, these data indicated that the high expression of *FBXW4* is a feature of higher-risk AML, which is more frequently seen in older AML adults and those with high-risk karyotypes, suggesting its clinical significance in predicting clinical outcomes in AML patients.

**Table 1:** Correlation of *FBXW4* expression with clinical and laboratorial parameters in AML patients.

	***FBXW4* high (TPM:1^**st**^ quartile, *n* = 39)**	***FBXW4* low (TPM: 2^**nd**^ to 4^**th**^ quartile, *n* = 116)**	***P***	***FBXW4* high (*Z* ≥ 1, *n* = 20)**	***FBXW4* low (*Z* < 1, *n* = 135)**	***P***
Sex, *n* (%)			0.058			0.889
Female	13 (33.3)	59 (50.9)		9 (45.0)	63 (47.7)	
Male	26 (66.7)	57 (49.1)		11 (55.0)	72 (52.3)	
Age, years			0.085			0.046
Median (range)	61 (21–88)	57 (18–82)		63.5 (38–88)	58 (18–82)	
WBC (× 10^9^/L)			0.990			0.892
Median (range)	27.6 (0.6–116.2)	18.3 (0.7–297.4)		26.9 (1.5–99.2)	19.6 (0.6–297.4)	
BM blasts (%)			0.543			0.814
Median (range)	75 (30–98)	70.5 (30–100)		69.5 (30–98)	72 (30–100)	
PB blasts (%)			0.891			0.476
Median (range)	48 (0–97)	42 (0–98)		33 (0–97)	45 (0–98)	
CRC, *n* (%)^*^			0.036			0.209
Favorable	0 (0.0)	17 (14.9)		0 (0)	17 (12.8)	
Intermediate	29 (76.3)	70 (61.4)		15 (78.9)	84 (63.1)	
Poor	9 (23.7)	27 (23.7)		4 (21.1)	32 (24.1)	
ELN risk stratification, *n* (%)^*^			0.129			
Favorable	4 (10.6)	30 (26.4)		1 (5.3)	33 (24.8)	0.137
Intermediate	17 (44.7)	42 (36.8)		10 (52.6)	49 (36.9)	
Adverse	17 (44.7)	42 (36.8)		8 (42.1)	51 (38.3)	
*FLT3, n* (%)			0.695			0.769
Wild type	30 (76.9)	82 (70.7)		14 (70.0)	98 (72.6)	
*FLT3-ITD*	7 (18.0)	24 (20.7)		5 (25.0)	26 (19.3)	
*FLT3-TKD*	2 (5.1)	10 (8.6)		1 (5.0)	11 (8.1)	
*TP53, n* (%)			0.856			0.780
Wild type	36 (92.3)	106 (91.4)		18 (90.0)	124 (91.9)	
Mutated	3 (7.7)	10 (8.6)		2 (10.0)	11 (8.1)	
Treatment, *n* (%)^#^			0.838			0.079
Intensive	31 (81.6)	93 (83.0)		13 (68.4)	111 (84.7)	
Non-intensive	7 (18.4)	19 (17.0)		6 (31.6)	20 (15.3)	
Allo-SCT, *n* (%)			0.208			0.100
Yes	13 (33.3)	52 (44.8)		5 (25.0)	60 (44.4)	
No	26 (66.7)	64 (55.2)		15 (75.0)	75 (55.6)	

* *Total patient number is 152 for three patients are lacking of evaluable cytogenetic information*.

# *Total patient number is 150 for five patients did not receive any treatment after diagnosis*.

*WBC, white blood cell; BM, bone marrow; PB, peripheral blood; CRC, cytogenetic risk classification; ELN, European Leukemia Net; Allo-SCT, allogeneic stem cell transplantation*.

As *FBXW5* is expressed the highest among FBXW proteins in AML cell lines and AML patients, we further analyzed the relationship between *FBXW5* expression and the clinical and laboratorial parameters. Unlike *FBXW4*, patients with age ≥60 and age <60 years old showed no significant difference in *FBXW5* expression (*P* > 0.05) (Figure 3C). Cytogenetic risk classification analysis results also exhibited a similar *FBXW5* expression in favorable-risk and intermediate/poor-risk AML patients (*P* > 0.05) (Figure 3D). However, when we dichotomized the patients into high *FBXW5* expression group (TPM: 1st quartile) and low *FBXW5* expression group (TPM: 2nd to 4th quartile) and explored their differences in clinical parameters, we found that the low *FBXW5* expression patients were relatively younger than the high *FBXW5* expression patients at diagnosis (median: 57 vs. 64, *P* = 0.017) (Table 2). Interestingly, the low *FBXW5* expression patients had a higher percentage to receive intensive chemotherapy (*P* = 0.005) and allo-SCT (*P* = 0.000) (Table 2). This may be partially because the patients with low *FBXW5* expression were younger and had better physical conditions to tolerate intensive chemotherapy and allo-SCT than those with high *FBXW5* expression. No significant differences were observed in sex, bone morrow blasts, *FLT3-ITD, FLT3-TKD*, and *TP53* mutation, cytogenetic risk classification, and ELN risk classification between the high and the low *FBXW5* expression patients (*P* > 0.05) (Table 2).

**Table 2:** Correlation of *FBXW5* expression with clinical and laboratorial parameters in AML patients.

	***FBXW5* high (TPM:1st quartile, *n* = 39)**	***FBXW5* low (TPM: 2nd to 4th quartile, *n* = 116)**	***P***	***FBXW5* high (*Z* ≥ 1, *n* = 24)**	***FBXW5* low (*Z* < 1, *n* = 131)**	***P***
Sex, *n* (%)			0.679			0.339
Female	17 (43.6)	55 (47.4)		9 (37.5)	63 (48.1)	
Male	22 (56.4)	61 (52.6)		15 (62.5)	68 (51.9)	
Age, years			0.017			0.102
Median (range)	64 (27–88)	57 (18–82)		62.5 (27–88)	57 (18–82)	
WBC (×10^9^/L)			0.368			0.095
Median (range)	22.9 (1.5–137.2)	21.5 (0.6–297.4)		24.7 (1.6–116.2)	17 (0.6–297.4)	
BM blasts (%)			0.965			0.724
Median (range)	74 (30–98)	70.5 (30–100)		74 (30–98)	71 (30–100)	
PB blasts (%)			0.009			0.180
Median (range)	22 (0–88)	48 (0–98)		27.5 (0–88)	46 (0–98)	
CRC, *n* (%)^*^			0.394			0.150
Favorable	2 (5.3)	15 (13.2)		0 (0)	17 (13.2)	
Intermediate	27 (71.0)	72 (63.1)		18 (78.3)	81 (62.8)	
Poor	9 (23.7)	27 (23.7)		5 (21.7)	31 (24.0)	
ELN risk stratification, *n* (%)^*^			0.236			
Favorable	6 (15.8)	28 (24.6)		2 (8.7)	32 (24.8)	0.102
Intermediate	19 (50.0)	40 (35.1)		13 (56.5)	46 (35.7	
Adverse	13 (34.2)	46 (40.3)		8 (34.8)	51 (39.5)	
*FLT3, n* (%)			0.603			0.087
Wild type	29 (74.4)	83 (71.6)		18 (75.0)	94 (71.8)	
*FLT3-ITD*	6 (15.4)	25 (21.5)		2 (8.3)	29 (22.1)	
*FLT3-TKD*	4 (10.2)	8 (6.9)		4 (16.7)	8 (6.1)	
*TP53, n* (%)			0.856			0.417
Wild type	36 (92.3)	106 (91.4)		23 (95.8)	119 (90.8)	
Mutated	3 (7.7)	10 (8.6)		1 (4.2)	12 (9.2)	
Treatment, *n* (%)^#^			0.005			0.182
Intensive	25 (69.4)	99 (87.6)		16 (72.7)	108 (84.4)	
Non-intensive	12 (30.6)	14 (12.4)		6 (27.3)	20 (15.6)	
Allo-SCT, *n* (%)			0.000			0.001
Yes	6 (15.4)	59 (50.9)		3 (12.5)	62 (47.3)	
No	33 (84.6)	57 (49.1)		21 (87.5)	69 (52.7)	

* *Total patient number is 152 for three patients are lacking of evaluable cytogenetic information*.

# *Total patient number is 150 for five patients did not receive any treatment after diagnosis*.

*WBC, white blood cell; BM, bone marrow; PB, peripheral blood; CRC, cytogenetic risk classification; ELN, European Leukemia Net; Allo-SCT, allogeneic stem cell transplantation*.

### High *FBXW4* Expression Is Associated With Poor Clinical Outcome in AML Patients

**Figure 4:**
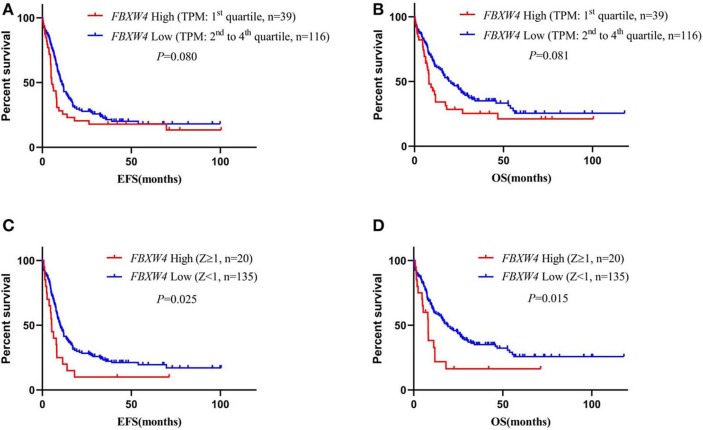
Survival analysis of AML patients according to *FBXW4* expression. **(A)** EFS of AML patients with high *FBXW4* expression (TPM: 1st quartile) and low *FBXW4* expression (TPM: 2nd to 4th quartile); **(B)** OS of AML patients with high *FBXW4* expression (TPM: 1st quartile) and low *FBXW4* expression (TPM: 2nd to 4th quartile); **(C)** EFS of AML patients with high *FBXW4* expression (*Z* ≥ 1) and low *FBXW4* expression (*Z* < 1); **(D)** OS of AML patients with high *FBXW4* expression (*Z* ≥ 1) and low *FBXW4* expression (*Z* < 1).

To best understand the significance of high *FBXW4* expression in the prognosis of AML patients, Kaplan–Meier survival curves were generated for overall survival (OS) and events-free survival (EFS) between patients with high and low *FBXW4* expression. Results showed that patients with high *FBXW4* expression (TPM: 1st quartile) had a shorter trend in EFS and OS compared with those with low *FBXW4* expression (TPM: 2nd to 4th quartile), although no statistical differences were detected (EFS: median: 5.3 vs. 10.9 months; *P* = 0.080; OS: median: 8.2 vs. 20.5 months; *P* = 0.081) (Figures 4A,B). However, when Z-scores = 1 was set as the cutoff, a significant difference was detected in EFS and OS between the high *FBXW4* expression (*Z* ≥ 1) patients and the low *FBXW4* expression (*Z* < 1) patients. The EFS and OS in patients with high *FBXW4* expression (*Z* ≥ 1) were significantly decreased when compared with those with low expression (*Z* < 1) (EFS: median: 5.3 vs. 10 months; *P* = 0.025; OS: median: 8.1 vs. 19 months; *P* = 0.015) (Figures 4C,D).

**Figure 5:**
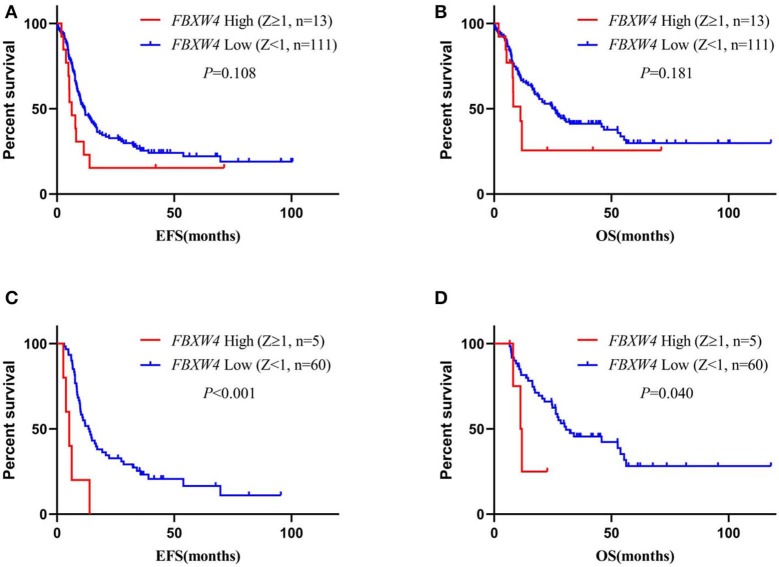
Survival analysis of AML patients who received intensive chemotherapy or intensive chemotherapy followed by allo-SCT according to *FBXW4* expression. **(A)** EFS of AML patients who received intensive chemotherapy with high *FBXW4* expression (*Z* ≥ 1) and low *FBXW4* expression (*Z* < 1); **(B)** OS of AML patients who received intensive chemotherapy with high *FBXW4* expression (*Z* ≥ 1) and low *FBXW4* expression (*Z* < 1); **(C)** EFS of AML patients who received intensive chemotherapy followed by allo-SCT with high *FBXW4* expression (*Z* ≥ 1) and low *FBXW4* expression (*Z* < 1); **(D)** OS of AML patients who received intensive chemotherapy followed by allo-SCT with high *FBXW4* expression (*Z* ≥ 1) and low *FBXW4* expression (*Z* < 1).

In multivariate survival analysis, the significant association of high *FBXW4* expression (*Z* ≥ 1) with shorter EFS and OS was not detected after adjusting for age, cytogenetic risk, allo-SCT status, and *TP53* and *FLT3-ITD* mutation (EFS: HR = 1.270, 95% CI: 0.742–2.174, *P* = 0.383; OS: HR = 1.367, 95% CI: 0.775–2.409, *P*= 0.280) (Table 3). As treatment options, especially intensive chemotherapy and allo-SCT, have profound positive influences on the prognosis of AML patients, we further screened out the 124 patients who received intensive chemotherapy and the 65 patients who received intensive chemotherapy followed by allo-SCT in the cohort to re-analyze the relationship between *FBXW4* expression and survival. As expected, in patients who received intensive chemotherapy, high *FBXW4* expression (*Z* ≥ 1) was associated with poorer EFS and OS, although there was no statistical significance (EFS: median: 6.3 vs. 11.8 months; *P* = 0.108; OS: median: 11.2 vs. 25.8 months; *P* = 0.181) (Figures 5A,B). In addition, for patients who received intensive chemotherapy followed by allo-SCT, the high *FBXW4* expression (*Z* ≥ 1) patients exhibited significantly shorter EFS and OS compared with the low *FBXW4* expression (*Z* < 1) patients (EFS: median: 5.2 vs. 13.6 months; *P* < 0.001; OS: median: 11.5 vs. 30.6 months; *P* = 0.040) (Figures 5C,D). Moreover, a multivariate survival analysis showed that high *FBXW4* expression (*Z* ≥ 1) was an independent risk factor of shorter EFS in AML patients who received intensive chemotherapy followed by allo-SCT (HR = 3.459, 95% CI: 1.140–10.494, *P*= 0.028) (Table 4). Taken together, these results indicated that high *FBXW4* expression is associated with poor clinical outcome in AML patients, and particularly it is an independent poor survival factor in patients with intensive chemotherapy and allo-SCT therapy.

**Table 3:** Cox proportional hazards model for overall survival and event-free survival in AML patients.

**Variables**	**OS**		**EFS**	
	**HR (95% CI)**	***P***	**HR (95% CI)**	***P***
Age (<60 vs. ≥ 60)	1.718 (1.111–2.657)	0.015	1.505 (1.020–2.221)	0.040
Cytogenetic risk		0.009		0.004
(favorable vs. intermediate/poor)	3.121 (1.322–7.371)		3.203 (1.441–7.118)	
Allo-SCT (yes vs. no)	0.498 (0.324–0.766)	0.001	0.703 (0.476–1.037)	0.076
*TP53* (WT vs. mutated)	2.857 (1.501–5.435)	0.001	2.371 (1.269–4.429)	0.007
*FLT3-ITD* (presence vs. absence)	1.381(0.840–2.268)	0.203	1.616 (1.024–2.550)	0.039
*FBXW4* (*Z* ≥ 1 vs. *Z* < 1)	1.367(0.775–2.409)	0.280	1.270 (0.742–2.174)	0.383

*HR, hazard ratio; CI, confidence interval*.

**Table 4:** Cox proportional hazards model for overall survival and event-free survival in AML patients who received intensive chemotherapy followed by allo-SCT.

**Variables**	**OS**		**EFS**	
	**HR (95% CI)**	***P***	**HR (95% CI)**	***P***
Age (<60 *vs*. ≥ 60)	0.925 (0.412–2.077)	0.850	0.857 (0.433–1.699)	0.659
Cytogenetic risk		0.922		0.411
(favorable vs. intermediate/poor)	0.940 (0.273–3.232)		0.664 (0.250–1.763)	
*TP53* (WT vs. mutated)	6.330 (1.605–24.958)	0.008	2.233 (0.622–8.018)	0.218
*FLT3-ITD* (presence vs. absence)	2.225 (0.985–5.025)	0.054	1.735 (0.816–3.686)	0.152
*FBXW4* (*Z* ≥ 1 vs. *Z* < 1)	2.654 (0.714–9.868)	0.145	3.459(1.140–10.494)	0.028

*HR, hazard ratio; CI, confidence interval*.

**Figure 6:**
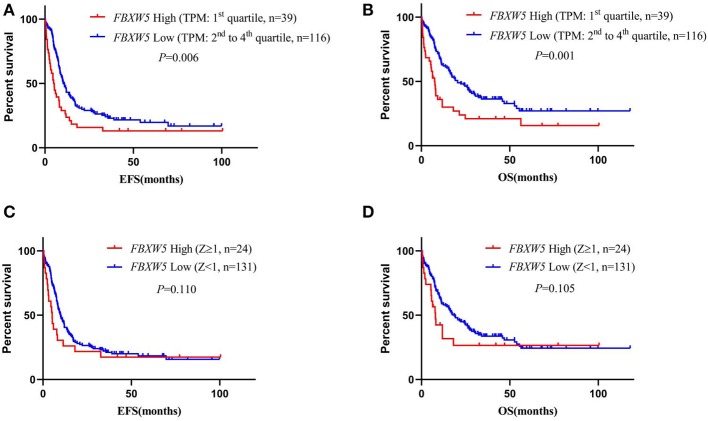
Survival analysis of AML patients according to *FBXW5* expression. **(A)** EFS of AML patients with *FBXW5* high expression (TPM: 1st quartile) and *FBXW5* low expression (TPM: 2nd to 4th quartile); **(B)** OS of AML patients with *FBXW5* high expression (TPM: 1st quartile) and *FBXW5* low expression (TPM: 2nd to 4th quartile); **(C)** EFS of AML patients with *FBXW5* high expression (*Z* ≥ 1) and *FBXW5* low expression (*Z* < 1); **(D)** OS of AML patients with *FBXW5* high expression (*Z* ≥ 1) and low *FBXW4* expression (*Z* < 1).

We also performed survival analysis according to *FBXW5* expression. As expected, low *FBXW5* expression (TPM: 2nd to 4th quartile) was associated with better EFS and OS in AML patients (EFS: median: 5.1 vs. 10.8 months; *P* = 0.006; OS: median: 7.5 vs. 20.5 months; *P* = 0.001) compared with that of high *FBXW5* expression (TPM: 1st quartile) (Figures 6A,B). This result was consistent with low *FBXW5* expression being associated with a high rate of intensive chemotherapy and allo-SCT as described above (Table 2). The survival differences no longer exist when *Z*-score = 1 was set as the cutoff to define the high and the low expression (*P* > 0.05) (Figures 6C,D). In patients who received intensive chemotherapy followed by allo-SCT, high *FBXW5* expression (*Z* ≥ 1) also predicted shorter EFS (median: 3.0 vs. 13.0 months; *P* < 0.001) (Figures S2A,B). However, as the sample size of the high *FBXW5* expression (*Z* ≥ 1) group is small (*n* = 3), the effect of *FBXW5* expression on survival needs to be further illustrated with a bigger sample size cohort in AML patients.

### Bioinformatic Analysis of *FBXW4* Function in AML

**Figure 7:**
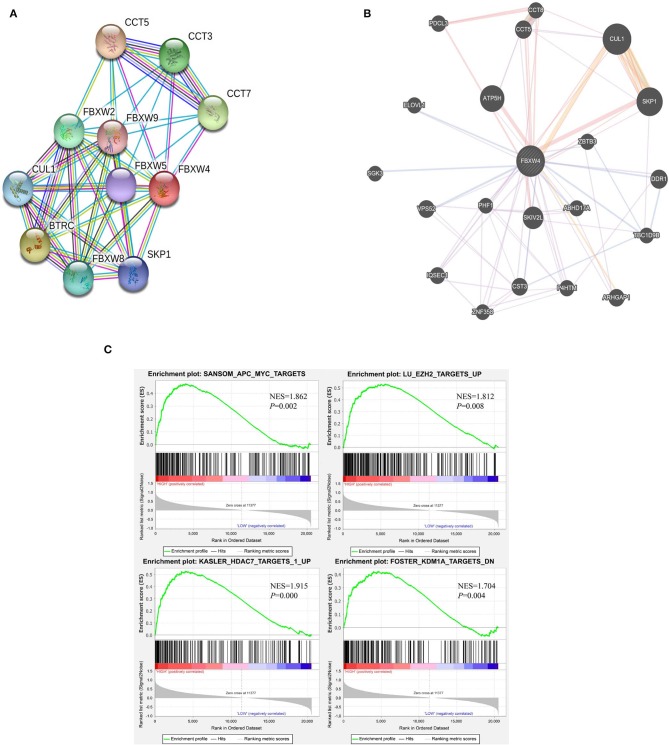
Potential biological functions of FBXW4 in AML. **(A)** Protein–protein interaction network of FBXW4 analyzed by STRING; **(B)** protein–protein interaction network of FBXW4 analyzed by GeneMANIA; **(C)** GSEA analysis of AML patients based on *FBXW4* expression.

As only a few proteins in the FBXW family are studied, there are no reports about FBXW4 function and its potential molecular mechanisms on oncogenesis. The protein–protein interactions of FBXW4 with other partners were analyzed using STRING and GeneMANIA online tools. Results showed that FBXW4 interacted with SKP1 and CUL1—two key components of Skp1-Cul1-F-box (SCF) E3 ubiquitin ligases (Figures 7A,B). The co-expression network analysis of FBXW4 was also conducted in the RNA sequencing data in the 173 AML patients used in the above analysis with cBioPortal dataset. The top 10 positively co-expressed genes of FBXW4 were identified as *PACS2, TRABD, PARP10, ARHGAP4, ACBD4, SIPA1, SH3GLB2, SH2B1, ZNF414*, and *MXD4* and the top negatively co-expressed genes were *ACBD3, ADSS, ERGIC2, CANX, MTM1, UBE2W, GMCL1, TXNDC9, VPS4B*, and *SSR1* (Table 5).

**Table 5:** Top 10 positively and negatively co-expressed genes of *FBXW4*.

**Correlated gene**	**Cytoband**	**Co-ex relationship**	**Spearman's correlation**	***p*-value**	***q*-value**
*PACS2*	14q32.33	Positively	0.69541466	2.51E-26	4.99E-22
*TRABD*	22q13.33	Positively	0.690220692	8.27E-26	8.20E-22
*PARP10*	8q24.3	Positively	0.67548243	2.12E-24	1.41E-20
*ARHGAP4*	Xq28	Positively	0.665354075	1.78E-23	8.82E-20
*ACBD4*	17q21.31	Positively	0.657951338	7.97E-23	3.16E-19
*SIPA1*	11q13.1	Positively	0.654405255	1.61E-22	5.33E-19
*SH3GLB2*	9q34.11	Positively	0.64989269	3.89E-22	1.10E-18
*SH2B1*	16p11.2	Positively	0.644951351	1.01E-21	2.50E-18
*ZNF414*	19p13.2	Positively	0.641590685	1.90E-21	4.19E-18
*MXD4*	4p16.3	Positively	0.637096662	4.39E-21	8.72E-18
*ACBD3*	1q42.12	Negatively	−0.593530832	7.60E-18	5.38E-15
*ADSS*	1q44	Negatively	−0.58895569	1.56E-17	9.67E-15
*ERGIC2*	12p11.22	Negatively	−0.558287868	1.46E-15	4.66E-13
*CANX*	5q35.3	Negatively	−0.551531769	3.72E-15	9.98E-13
*MTM1*	Xq28	Negatively	−0.550263986	4.43E-15	1.12E-12
*UBE2W*	8q21.11	Negatively	−0.549014745	5.25E-15	1.24E-12
*GMCL1*	2p13.3	Negatively	−0.541832189	1.38E-14	2.55E-12
*TXNDC9*	2q11.2	Negatively	−0.533319273	4.21E-14	6.06E-12
*VPS4B*	18q21.33	Negatively	−0.530869462	5.77E-14	7.60E-12
*SSR1*	6p24.3	Negatively	−0.52689924	9.57E-14	1.19E-11

GSEA analysis of RNA-seq data was performed to further explore the involved biological pathways and cofactors of FBXW4 in AML. High *FBXW4* expression was defined as TPM in the 1st quartile, and low *FBXW4* expression was defined as TPM in the 4th quartile. The results showed that in the patients with high *FBXW4* expression, the gene sets were significantly enriched in APC-MYC (WNT signaling pathway) [normalized enrichment score (NES) = 1.862, *P* = 0.002], EZH2 targets (NES = 1.862, *P* = 0.008), HDAC7 targets (NES = 1.915, *P* = 0.000), and KDM1A targets (NES = 1.704, *P* = 0.004) (Figure 7C). As EZH2, HDAC7, and KDM1A are all key epigenetic regulators with function on gene transcription and tumorigenesis, our data suggested that FBXW4 may be involved in epigenomic regulation by inducing the ubiquitination of those epigenetic proteins in AML.

Taken together, these results revealed that FBXW4 may play an important role in oncogenesis by substrate-mediated degradation through the assembly of SCF E3 ubiquitin ligases and may also be involved in multi-oncogenic pathways through a complex regulation network.

## Discussion

Ubiquitin proteasome-mediated protein degradation plays vital roles in various cellular processes such as proliferation, differentiation, and apoptosis (9, 17, 18, 21). FBXW proteins belong to the ubiquitin proteasome family. With several powerful publicly available datasets, herein we identified that the high expression of *FBXW4*, the less studied member of FBXW proteins, is associated with older age, poorer cytogenetic risk classification, and shorter survival in AML patients. Our data revealed that high *FBXW4* expression may have the potential to be a potent prognostic biomarker for AML.

Functional studies of FBXW proteins in cancers are well-known to be involved in mediating substrate protein degradation. FBXW proteins can play either tumor suppressor or tumorigenesis roles in different cancers based on the substrate proteins' function in cellular processes. In hematopoietic malignancies, FBXW7 has been identified as a tumor suppressor for its ability to mediate proto-oncogene protein degradation in T-cell acute lymphoblastic leukemia (16). We found that *FBXW4* was expressed higher in AML patients compared with that in normal controls. High *FBXW4* expression was significantly associated with older age and poorer cytogenetic risk classification in AML. A survival analysis showed that high *FBXW4* expression had a direct correlation with shorter OS and EFS compared with low *FBXW4* expression in AML patients. A multivariate survival analysis showed that high *FBXW4* expression is an independent risk factor of the poor survival in patients with intensive chemotherapy and allo-SCT. Our data not only indicated that high *FBXW4* expression is a feature of higher-risk AML but also suggested that FBXW4 may play oncogenic roles in AML.

We observed that high *FBXW4* expression did not have statistical significance as an independent poor survival factor if the treatment conditions in AML patients were not considered; however, it became an independent risk factor of poor survival in patients with intensive chemotherapy and allo-SCT. The possible reason is that in the AML patients, without the considering the treatment options, high *FBXW4* expression was strongly associated with poorer cytogenetic risk and older age; this association may partly interfere with the effect of high *FBXW4* expression as an independent survival factor. The patients who received intensive chemotherapy followed by allo-SCT usually show greater consistency in clinical characters and physical conditions, which may facilitate the role of high *FBXW4* expression as an independent risk factor of shorter EFS in these patients.

Moreover, we observed that high *FBXW4* expression is associated with both older age and poorer cytogenetic risk classification in AML. We noticed that there was an overlap in the older AML patients and those with poor-risk karyotypes in our analyzed data; thus, our data emphasized that the high *FBXW4* expression is a more critical feature in older AML adults with higher-risk karyotypes.

Furthermore, although *FBXW5* is expressed the highest in AML cell lines and in AML patients, we did not find an expression difference of *FBXW5* between the AML patients and the normal controls. A survival analysis also showed that low *FBXW5* expression was associated with a high rate of intensive chemotherapy and allo-SCT and associated with better survival in AML patients. High *FBXW5* expression was associated with poor survival in patients who received intensive chemotherapy followed by allo-SCT. However, the relationship of *FBXWB5* expression with survival in AML needs to be further explored in the future with a much bigger cohort study.

Previous work on FBXW4 mainly focused on its role in split hand and foot malformation (31, 32). Our analysis data here strongly suggest the role of FBXW4 in the oncogenesis of AML. Protein–protein interaction prediction results showed that FBXW4 interacts with two key components of SCF E3 ubiquitin ligases, SKP1 and CUL1. It is reported that FBXW7 acts as a tumor suppressor through the assembly of SCF E3 ubiquitin ligases as well. As WD repeat domain is responsible for substrate binding, the differences between FBXW7 and FBXW4 in WD repeat domain amounts (seven for FBXW7 and six for FBXW4) and space structures indicate that they possibly have different preferences in substrate binding. This may explain why FBXW7 functions as a tumor suppressor, while our data revealed that FBXW4 has oncogenic effects in AML. Also, we analyzed *FBXW4* co-expression network for the first time, and these results suggest that *FBXW4* may work together with other signaling pathways to exert its oncogenic effect in AML, which also provides hints about its underlying molecular mechanism in oncogenesis. Moreover, GSEA analysis indicated that high *FBXW4* expression was involved in several epigenetic regulation gene sets in AML patients, providing a potential and interesting direction for further exploration of its biological functions.

High *FBXW4* expression is associated with poor survival in AML patients. It is not only used as a prognostic factor particularly for the patient with intensive chemotherapy and allo-SCT but also it has the potential for developing new targeting therapy. FBXW4 has six conserved WD repeat domains which are responsible for substrate binding. Drugs targeting another WD repeat domain-containing protein—WDR5—are available and exhibited strong tumor suppression ability in several human cancers including hematologic malignancies (33–35). Another drug (MLN4924, Pevonedistat), which can inhibit cellular cullin RING ubiquitin ligases, has also been tested as an anti-tumor drug in several clinical trials (36, 37). These inspiring results give us convincing evidences and strong confidence that the WD repeat domain and the ubiquitin ligases system are efficient therapeutic targets, and the development of new drugs targeting other WD repeat domain-containing proteins like FBXW4 has broad prospects in the future.

In summary, we systemically analyzed the transcriptional expression profile of FBXW proteins in AML cell lines and patients. Our results indicated that *FBXW4* is aberrantly expressed in AML patients, and its high expression is associated with high risk factors and poor prognosis; particularly, it is an independent poor survival factor in patients with intensive chemotherapy and allo-SCT therapy. FBXW4 may mediate substrate degradation through the assembly of SCF E3 ubiquitin ligases. Further work on detecting its interacting substrate proteins will provide more information about its function and underlying molecular mechanism to further illustrate its role in human cancers.

## Data Availability Statement

Publicly available datasets were analyzed in this study. This data can be found here: https://www.broadinstitute.org/ccle.

## Author Contributions

QH participated in manuscript writing and carried out data collection and analysis. QZ and HS carried out data collection and analysis. YB participated in manuscript writing. CS and ZG participated in manuscript writing and took charge of overall instruction.

### Conflict of Interest

The authors declare that the research was conducted in the absence of any commercial or financial relationships that could be construed as a potential conflict of interest.

## Supplementary Material

Figure S1 Expression differences of FBXW1, FBXW2, FBXW7, FBXW8, FBXW9, FBXW10, FBXW11, and FBXW12 between 173 *de novo* AML patients and 70 normal controls.

Figure S2 Survival analysis of AML patients who received intensive chemotherapy followed by allo-SCT according to FBXW5 expression. **(A)** EFS of AML patients who received intensive chemotherapy followed by allo-SCT with FBXW5 high expression (Z ≥ 1) and FBXW5 low expression (Z < 1); **(B)** OS of AML patients who received intensive chemotherapy followed by allo-SCT with FBXW5 high expression (Z ≥ 1) and FBXW5 low expression (Z < 1).

## References

[1] Dohner H Weisdorf DJ Bloomfield CD. Acute myeloid leukemia. N Engl J Med. (2015) 373:1136–52. 10.1056/NEJMra1406184 26376137

[2] De Kouchkovsky I Abdul-Hay M. 'Acute myeloid leukemia: a comprehensive review and 2016 update'. Blood Cancer J. (2016) 6:e441. 10.1038/bcj.2016.50 27367478 PMC5030376

[3] Dohner H Estey E Grimwade D Amadori S Appelbaum FR Buchner T. Diagnosis and management of AML in adults: 2017 ELN recommendations from an international expert panel. Blood. (2017) 129:424–47. 10.1182/blood-2016-08-733196 27895058 PMC5291965

[4] Levis M Brown P Smith BD Stine A Pham R Stone R. Plasma inhibitory activity (PIA): a pharmacodynamic assay reveals insights into the basis for cytotoxic response to FLT3 inhibitors. Blood. (2006) 108:3477–83. 10.1182/blood-2006-04-015743 16857987 PMC1895426

[5] Shih AH Abdel-Wahab O Patel JP Levine RL. The role of mutations in epigenetic regulators in myeloid malignancies. Nat Rev Cancer. (2012) 12:599–612. 10.1038/nrc3343 22898539

[6] Bullinger L Dohner K Dohner H. Genomics of acute myeloid leukemia diagnosis and pathways. J Clin Oncol. (2017) 35:934–46. 10.1200/JCO.2016.71.2208 28297624

[7] Yamaura T Nakatani T Uda K Ogura H Shin W Kurokawa N. A novel irreversible FLT3 inhibitor, FF-10101, shows excellent efficacy against AML cells with FLT3 mutations. Blood. (2018) 131:426–38. 10.1182/blood-2017-05-786657 29187377

[8] Akhoondi S Sun D von der Lehr N Apostolidou S Klotz K Maljukova A. FBXW7/hCDC4 is a general tumor suppressor in human cancer. Cancer Res. (2007) 67:9006–12. 10.1158/0008-5472.CAN-07-1320 17909001

[9] Welcker M Clurman BE. FBW7 ubiquitin ligase: a tumour suppressor at the crossroads of cell division, growth and differentiation. Nat Rev Cancer. (2008) 8:83–93. 10.1038/nrc2290 18094723

[10] Koepp DM Schaefer LK Ye X Keyomarsi K Chu C Harper JW. Phosphorylation-dependent ubiquitination of cyclin E by the SCFFbw7 ubiquitin ligase. Science. (2001) 294:173–7. 10.1126/science.1065203 11533444

[11] Oberg C Li J Pauley A Wolf E Gurney M Lendahl U. The Notch intracellular domain is ubiquitinated and negatively regulated by the mammalian Sel-10 homolog. J Biol Chem. (2001) 276:35847–53. 10.1074/jbc.M103992200 11461910

[12] Strohmaier H Spruck CH Kaiser P Won KA Sangfelt O Reed SI. Human F-box protein hCdc4 targets cyclin E for proteolysis and is mutated in a breast cancer cell line. Nature. (2001) 413:316–22. 10.1038/35095076 11565034

[13] Welcker M Orian A Jin J Grim JE Harper JW Eisenman RN. The Fbw7 tumor suppressor regulates glycogen synthase kinase 3 phosphorylation-dependent c-Myc protein degradation. Proc Natl Acad Sci USA. (2004) 101:9085–90. 10.1073/pnas.0402770101 15150404 PMC428477

[14] Yada M Hatakeyama S Kamura T Nishiyama M Tsunematsu R Imaki H. Phosphorylation-dependent degradation of c-Myc is mediated by the F-box protein Fbw7. Embo J. (2004) 23:2116–25. 10.1038/sj.emboj.7600217 15103331 PMC424394

[15] Calhoun ES Jones JB Ashfaq R Adsay V Baker SJ Valentine V. BRAF and FBXW7 (CDC4, FBW7, AGO, SEL10) mutations in distinct subsets of pancreatic cancer: potential therapeutic targets. Am J Pathol. (2003) 163:1255–60. 10.1016/S0002-9440(10)63485-2 14507635 PMC1868306

[16] Yeh CH Bellon M Pancewicz-Wojtkiewicz J Nicot C. Oncogenic mutations in the FBXW7 gene of adult T-cell leukemia patients. Proc Natl Acad Sci USA. (2016) 113:6731–6. 10.1073/pnas.1601537113 27247421 PMC4914202

[17] Xu J Zhou W Yang F Chen G Li H Zhao Y. The beta-TrCP-FBXW2-SKP2 axis regulates lung cancer cell growth with FBXW2 acting as a tumour suppressor. Nat Commun. (2017) 8:14002. 10.1038/ncomms14002 28090088 PMC5241824

[18] Kim TY Jackson S Xiong Y Whitsett TG Lobello JR Weiss GJ. CRL4A-FBXW5-mediated degradation of DLC1 Rho GTPase-activating protein tumor suppressor promotes non-small cell lung cancer cell growth. Proc Natl Acad Sci USA. (2013) 110:16868–73. 10.1073/pnas.1306358110 24082123 PMC3801067

[19] Okabe H Lee SH Phuchareon J Albertson DG McCormick F Tetsu O. A critical role for FBXW8 and MAPK in cyclin D1 degradation and cancer cell proliferation. PLoS ONE. (2006) 1:e128. 10.1371/journal.pone.0000128 17205132 PMC1762433

[20] Hanahan D Weinberg RA. Hallmarks of cancer: the next generation. Cell. (2011) 144:646–74. 10.1016/j.cell.2011.02.013 21376230

[21] Wang L Feng W Yang X Yang F Wang R Ren Q. Fbxw11 promotes the proliferation of lymphocytic leukemia cells through the concomitant activation of NF-kappaB and beta-catenin/TCF signaling pathways. Cell Death Dis. (2018) 9:427. 10.1038/s41419-018-0440-1 29555946 PMC5859049

[22] Vazquez-Dominguez I Gonzalez-Sanchez L Lopez-Nieva P Fernandez-Navarro P Villa-Morales M Cobos-Fernandez MA. Downregulation of specific FBXW7 isoforms with differential effects in T-cell lymphoblastic lymphoma. Oncogene. (2019) 38:4620–36. 10.1038/s41388-019-0746-1 30742097

[23] Craddock C Labopin M Robin M Finke J Chevallier P Yakoub-Agha I. Clinical activity of azacitidine in patients who relapse after allogeneic stem cell transplantation for acute myeloid leukemia. Haematologica. (2016) 101:879–83. 10.3324/haematol.2015.140996 27081178 PMC5004468

[24] Barretina J Caponigro G Stransky N Venkatesan K Margolin AA Kim S. The Cancer Cell Line Encyclopedia enables predictive modelling of anticancer drug sensitivity. Nature. (2012) 483:603–7. 10.1038/nature11003 22460905 PMC3320027

[25] Tang Z Li C Kang B Gao G Li C Zhang Z. GEPIA: a web server for cancer and normal gene expression profiling and interactive analyses. Nucleic Acids Res. (2017) 45:W98–102. 10.1093/nar/gkx247 28407145 PMC5570223

[26] Ley TJ Miller C Ding L Raphael BJ Mungall AJ Robertson A. Genomic and epigenomic landscapes of adult de novo acute myeloid leukemia. N Engl J Med. (2013) 368:2059–74. 10.1056/NEJMoa1301689 23634996 PMC3767041

[27] Cerami E Gao J Dogrusoz U Gross BE Sumer SO Aksoy BA. The cBio cancer genomics portal: an open platform for exploring multidimensional cancer genomics data. Cancer Discov. (2012) 2:401–4. 10.1158/2159-8290.CD-12-0095 22588877 PMC3956037

[28] Gao J Aksoy BA Dogrusoz U Dresdner G Gross B Sumer SO. Integrative analysis of complex cancer genomics and clinical profiles using the cBioPortal. Sci Signal. (2013) 6:pl1. 10.1126/scisignal.2004088 23550210 PMC4160307

[29] Warde-Farley D Donaldson SL Comes O Zuberi K Badrawi R Chao P. The GeneMANIA prediction server: biological network integration for gene prioritization and predicting gene function. Nucleic Acids Res. (2010) 38(Web Server issue):W214–20. 10.1093/nar/gkq537 20576703 PMC2896186

[30] Szklarczyk D Gable AL Lyon D Junge A Wyder S Huerta-Cepas J. STRING v11: protein-protein association networks with increased coverage, supporting functional discovery in genome-wide experimental datasets. Nucleic Acids Res. (2019) 47:D607–13. 10.1093/nar/gky1131 30476243 PMC6323986

[31] Friedli M Nikolaev S Lyle R Arcangeli M Duboule D Spitz F. Characterization of mouse dactylaplasia mutations: a model for human ectrodactyly SHFM3. Mamm Genome. (2008) 19:272–8. 10.1007/s00335-008-9106-0 18392654

[32] Dimitrov BI de Ravel T Van Driessche J de Die-Smulders C Toutain A Vermeesch JR. Distal limb deficiencies, micrognathia syndrome, and syndromic forms of split hand foot malformation (SHFM) are caused by chromosome 10q genomic rearrangements. J Med Genet. (2010) 47:103–11. 10.1136/jmg.2008.065888 19584065

[33] Malek R Gajula RP Williams RD Nghiem B Simons BW Nugent K. TWIST1-WDR5-Hottip regulates Hoxa9 chromatin to facilitate prostate cancer metastasis. Cancer Res. (2017) 77:3181–93. 10.1158/0008-5472.CAN-16-2797 28484075 PMC5489316

[34] Zhang X Zheng X Yang H Yan J Fu X Wei R. Piribedil disrupts the MLL1-WDR5 interaction and sensitizes MLL-rearranged acute myeloid leukemia (AML) to doxorubicin-induced apoptosis. Cancer Lett. (2018) 431:150–60. 10.1016/j.canlet.2018.05.034 29857126

[35] Punzi S Balestrieri C D'Alesio C Bossi D Dellino GI Gatti E. WDR5 inhibition halts metastasis dissemination by repressing the mesenchymal phenotype of breast cancer cells. Breast Cancer Res. (2019) 21:123. 10.1186/s13058-019-1216-y 31752957 PMC6873410

[36] Swords RT Coutre S Maris MB Zeidner JF Foran JM Cruz J. Pevonedistat, a first-in-class NEDD8-activating enzyme inhibitor, combined with azacitidine in patients with AML. Blood. (2018) 131:1415–24. 10.1182/blood-2017-09-805895 29348128 PMC5909884

[37] Lockhart AC Bauer TM Aggarwal C Lee CB Harvey RD Cohen RB Phase Ib study of pevonedistat, a NEDD8-activating enzyme inhibitor, in combination with docetaxel, carboplatin and paclitaxel, or gemcitabine, in patients with advanced solid tumors. Invest New Drugs. (2019) 37:87–97. 10.1007/s10637-018-0610-0 29781056 PMC6510847

