# Supplementary figures and images for: FBXW4 Is Highly Expressed and Associated With Poor Survival in Acute Myeloid Leukemia
Source: Front Oncol. 2020 Feb 27;10:149. doi: 10.3389/fonc.2020.00149 (PMC7056870; doi:10.3389/fonc.2020.00149)

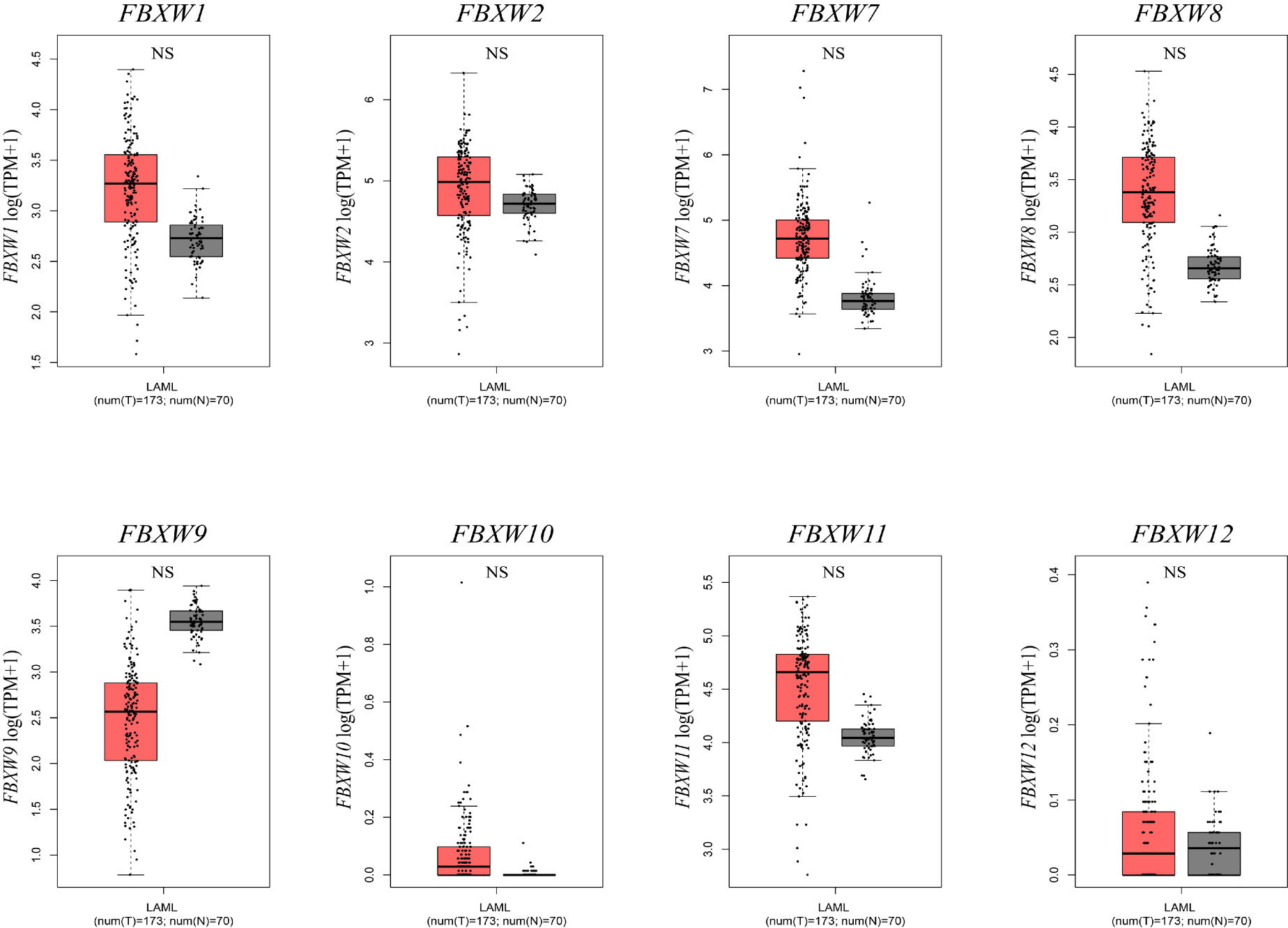

Supplement: Figure S1 — Expression differences of FBXW1, FBXW2, FBXW7, FBXW8, FBXW9, FBXW10, FBXW11, and FBXW12 between 173 de novo AML patients and 70 normal controls. [file Image_1.jpeg]

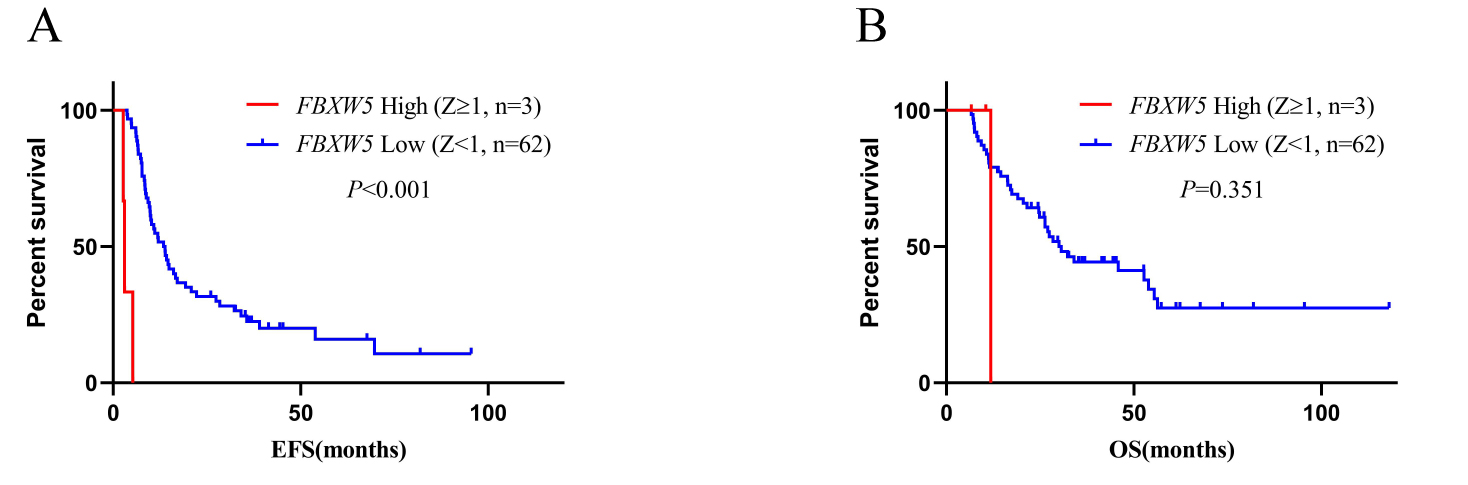

Supplement: Figure S2 — Survival analysis of AML patients who received intensive chemotherapy followed by allo-SCT according to FBXW5 expression. (A) EFS of AML patients who received intensive chemotherapy followed by allo-SCT with FBXW5 high expression (Z ≥ 1) and FBXW5 low expression (Z < 1); (B) OS of AML patients who received intensive chemotherapy followed by allo-SCT with FBXW5 high expression (Z ≥ 1) and FBXW5 low expression (Z < 1). [file Image_2.jpeg]
